# Diagnostic and prognostic evaluation of soluble CD14 subtype for sepsis in critically ill patients: a preliminary study

**DOI:** 10.1186/cc14053

**Published:** 2014-12-03

**Authors:** A Foca, C Peronace, V Marano, GS Barreca, AG Lamberti, N Marascio, A Giancotti, R Puccio, MC Liberto

**Affiliations:** 1 Department of Health Sciences, Institute of Microbiology, University 'Magna Graecia' of Catanzaro, Italy

## Introduction

Sepsis, a leading cause of death in critical care patients, is the result of complex interactions between the infecting microorganisms and the host responses that influence clinical outcomes [1]. Reliable biochemical markers that enable early diagnosis are still needed. A new marker, presepsin (soluble CD14 subtype, sCD14-ST) is a circulating fragment of 13 kDa of soluble CD14 that originates from the cleavage of the CD14 expressed on the surface membranes of monocytes/macrophages. sCD14-ST has been shown to increase significantly in patients with sepsis [2]. In our study we compare diagnostic performance of presepsin, C-reactive protein (CRP) and procalcitonin (PCT).

## Methods

Critical patients with suspected sepsis admitted to the Unit of Intensive Care of the University Hospital of Catanzaro (Italy) were recruited into this study; healthy volunteers were also included as controls. Plasma samples in EDTA from each patient were collected at multiple time points; samples were tested for CRP, PCT and presepsin. Blood cultures were also evaluated and processed by a BacT/Alert 3D system (bioMérieux, Italy); CRP was measured by immunonephelometry (Siemens Healthcare Diagnostic, Italy) and PCT was assayed by an enzyme-linked fluorescent assay (VIDAS BRAHMS PCT, bioMérieux, Italy); presepsin levels were measured by rapid automated PATHFAST immunoanalyzer (kindly provided by GEPA SRL, Italy), based on chemiluminescent enzyme immunoassay. A statistical analysis was carried out by Mann-Whitney test.

## Results

Presepsin and PCT levels were significantly higher in culture-positive subjects versus negative controls; such difference was found even at the admission time. The presepsin values in worsening/dead patients exhibited a significantly higher level at admission time. On the contrary, in the same group of patients, PCT exhibited a decrease of its level. In poor prognosis patients CRP showed a quite irregular kinetic, although in such a group the admission value was higher than the same marker in live subjects.

## Conclusion

In this preliminary study, presepsin and PCT levels exhibited substantial higher values in culture-positive patients. The kinetic curves of presepsin, obtained from both survival and worsening/dead subjects, revealed the optimal performance of this biomarker, particularly in severely ill patients, as also shown in other studies. During sepsis, increase of presepsin levels may be a more reliable marker, indicating an unfavorable outcome [3,4]. Furthermore, high presepsin levels could alert clinicians not to suspend antibiotic treatments even after clinical symptoms have improved and PCT levels have returned to normal.
